# User-driven development and evaluation of an agentic framework for analysis of large pathway diagrams

**DOI:** 10.1093/bioadv/vbag277

**Published:** 2026-09-21

**Authors:** Marie Corradi, Ivo Djidrovski, Luiz Ladeira, Bernard Staumont, Anouk Verhoeven, Julen Sanz Serrano, Adrien Rougny, Ahmad Vaez, Ahmed Hemedan, Alexander Mazein, Anna Niarakis, Arthur de Carvalho e Silva, Charles Auffray, Egon Willighagen, Eliska Kuchovska, Falk Schreiber, Irina Balaur, Laurence Calzone, Lisa Matthews, Lorenzo Veschini, Marc E Gillespie, Martina Kutmon, Matthias König, Matti van Welzen, Noriko Hiroi, Oxana Lopata, Pierre Klemmer, Rupert Overall, Tim Hofer, Venkata Satagopam, Reinhard Schneider, Marc Teunis, Liesbet Geris, Marek Ostaszewski

**Affiliations:** Innovative Testing in Life Sciences and Chemistry, HU—University of Applied Sciences Utrecht, 3584 CS Utrecht, Netherlands; Institute of Risk Assessment Sciences (IRAS), Utrecht University, 3584 CM Utrecht, Netherlands; Biomechanics Research Unit, GIGA Institute, University of Liège, 4000 Liège, Belgium; Biomechanics Research Unit, GIGA Institute, University of Liège, 4000 Liège, Belgium; Department of Pharmaceutical and Pharmacological Sciences, Vrije Universiteit Brussel, 1090 Jette, Belgium; Department of Pharmaceutical and Pharmacological Sciences, Vrije Universiteit Brussel, 1090 Jette, Belgium; Luxembourg Centre for Systems Biomedicine, University of Luxembourg, L-4362 Esch-sur-Alzette, Luxembourg; Unit of Genetic Epidemiology & Bioinformatics, Department of Epidemiology, University Medical Center Groningen, 9700 RB Groningen, Netherlands; Department of Bioinformatics, Isfahan University of Medical Sciences, 8174673461 Isfahan, Iran; Medical Image and Signal Processing Research Center, Isfahan University of Medical Sciences, 8174673461 Isfahan, Iran; Transversal Translational Medicine, Luxembourg Institute of Health, L-1445 Strassen, Luxembourg; Department of Cancer Research, Luxembourg Institute of Health, L-1445 Strassen, Luxembourg; Faculty of Science and Engineering, Department of Biology and Geosciences, University of Toulouse, 31062 Toulouse, France; Computational Systems Biology Hub, Center for Integrative Biology, University of Toulouse, CNRS, 31400 Toulouse, France; Centre for Environmental Research and Justice (CERJ), School of Biosciences, University of Birmingham, Birmingham, B15 2TT, United Kingdom; European Institute for Systems Biology and Medicine, 69390 Vourles, France; Translational Genomics, NUTRIM, FHML, Maastricht University, 6200 MD Maastricht, Netherlands; IUF - Leibniz Research Institute for Environmental Medicine, 40225 Düsseldorf, Germany; Department of Computer and Information Science, University of Konstanz, 78457 Konstanz, Germany; Faculty of IT, Monash University, Clayton Victoria 3800, Australia; Luxembourg National Data Service, L-4362 Esch-sur-Alzette, Luxembourg; Computational Systems Biology of Cancer, Institut Curie, U1331 INSERM, Mines ParisTech, PSL, 75005 Paris, France; Biochemistry and Molecular Pharmacology, NYU Grossman School of Medicine, New York, NY 10016, United States; Biocomplexity Institute, Department of Intelligent Systems Engineering, Indiana University at Bloomington, Bloomington, IN 47408, United States; Department of Pharmaceutical Sciences, St. John’s University, Queens, NY 11439, United States; Maastricht Centre for Systems Biology and Bioinformatics (MaCSBio), Maastricht University, 6229 EN Maastricht, Netherlands; Institute of Biology, Humboldt University of Berlin, 10099 Berlin, Germany; Institute of Structural Mechanics and Dynamics in Aerospace Engineering, University of Stuttgart, 70569 Stuttgart, Germany; First Department of Medicine, Campus Lübeck, University Hospital Schleswig-Holstein, 23538 Lübeck, Germany; Department of Systems Biology and Bioinformatics, University of Rostock, 18057 Rostock, Germany; Department of Electrical and Electronic Engineering, Kanagawa Institute of Technology, Kanagawa 243-0292, Japan; School of Medicine, Keio University, Tokyo 108-0073, Japan; Luxembourg Centre for Systems Biomedicine, University of Luxembourg, L-4362 Esch-sur-Alzette, Luxembourg; Luxembourg Centre for Systems Biomedicine, University of Luxembourg, L-4362 Esch-sur-Alzette, Luxembourg; Institute of Biology, Humboldt University of Berlin, 10099 Berlin, Germany; Department of Chemical Toxicology, Norwegian Institute of Public Health, N-0213 Oslo, Norway; Luxembourg Centre for Systems Biomedicine, University of Luxembourg, L-4362 Esch-sur-Alzette, Luxembourg; Luxembourg Centre for Systems Biomedicine, University of Luxembourg, L-4362 Esch-sur-Alzette, Luxembourg; Innovative Testing in Life Sciences and Chemistry, HU—University of Applied Sciences Utrecht, 3584 CS Utrecht, Netherlands; Biomechanics Research Unit, GIGA Institute, University of Liège, 4000 Liège, Belgium; Luxembourg Centre for Systems Biomedicine, University of Luxembourg, L-4362 Esch-sur-Alzette, Luxembourg

## Abstract

*Summary:* As biomedical knowledge keeps growing, resources storing available information multiply and grow in size and complexity. Such resources can be in the format of molecular interaction maps, which represent cellular and molecular processes under normal or pathological conditions. However, these maps can be complex and hard to navigate, especially to novice users. Large language models (LLMs), particularly in the form of agentic frameworks, have emerged as a promising technology to support this exploration. In this article, we describe a user-driven process of prototyping, development, and user testing of Llemy, an LLM-based system for exploring these molecular interaction maps. By involving domain experts from the very first prototyping in the form of a hackathon and collecting both fine-grained and general feedback on more refined versions, we were able to evaluate the perceived utility and quality of the developed system, in particular for summarizing maps and pathways, as well as prioritize the development of future features. We recommend continued user-driven development and benchmarking to keep the community engaged. This will also facilitate the transition towards open-weight LLMs to support the needs of the open research environment in an ever-changing technology landscape.

## 1 Introduction

The ecosystem of biomedical knowledge repositories continues to grow in size and complexity. Structured information available in databases of pathway diagrams, knowledge graphs, or interaction databases can support hypothesis generation, inform experimental design, and facilitate interpretation of omics dat (Mazein *et al.* 2024). However, navigating resources with different formats, interfaces, and granularities is a challenging and time-consuming task. Additionally, the sheer volume of these knowledge collections poses a challenge for navigating their individual components and relationships.

One such resource – disease maps – consists of comprehensive graphical representations of cellular and molecular processes associated with specific organ functions under normal or pathological conditions (Mazein *et al.* 2018, Staumont *et al.* 2025). Such maps are manually curated, provide information about source literature used for their construction, and follow the Systems Biology Graphical Notation (Le Novère *et al.* 2009) and Systems Biology Markup Language (SBML) (Keating *et al.* 2020) standards. They are hosted using the MINERVA Platform (Hoksza *et al.* 2019) which provides functionalities for visual exploration and programmatic access. Currently, a number of disease and physiological maps are available, describing pathways of neurodegeneration (Fujita *et al.* 2014), chronic immune and allergic diseases (Mazein *et al.* 2021, Acencio *et al.* 2023, 2026, Lopata *et al.* 2025), mechanisms of inflammation and viral infection (Serhan *et al.* 2020, Ostaszewski *et al.* 2021), and physiology of liver metabolism (Ladeira *et al.* 2025) or adverse outcome pathways (Mazein *et al.* 2025). Importantly, molecular interaction maps are distributed and differ in scope and granularity, and can benefit from unified access interfaces to reduce barriers to access and interpretation. This will facilitate visualization, analysis, and modelling of complex molecular mechanisms represented by these maps (Hoch *et al.* 2022, 2023, Zerrouk *et al.* 2022, 2024, Hemedan *et al.* 2024).

Large language models (LLMs) have emerged as a promising technology for summarizing, analyzing, and explaining structured knowledge. They range from general-purpose foundational models to applications in specialized tasks like building and exploring knowledge graphs or data analysis and hypothesis generation. Direct retrieval of knowledge from LLMs has been evaluated in, e.g. assisting pathway enrichment analysis (Azam *et al.* 2024), retrieving ageing-relevant knowledge under different hyperparameter configurations (Shakhbazau 2026), or searching for drug combinations in Alzheimer’s disease (Wang *et al.* 2026). For processing structured knowledge, LLM-based workflows have been proposed to generate knowledge graphs from the literature (James *et al.* 2026) and to process them for prioritizing experimental results (Feng *et al.* 2025) and exploring clinically valid hypotheses (Khan *et al.* 2025). Additionally, frameworks have been proposed to convert biological databases into structured knowledge graphs (Lobentanzer *et al.* 2023) and to access information via chatbot interfaces (Lobentanzer *et al.* 2025). In parallel to specific use cases, LLM-supported architectures were proposed to assist with access to bioinformatic databases and summary of results (Sokolova *et al.* 2025), supporting collaborative construction of scientific hypotheses (Jiang *et al.* 2026), or comprehensive analysis of omics data (Nagarajan *et al.* 2025). Finally, LLMs were evaluated for their support in curation and annotation of bioinformatic knowledge (Devkota *et al.* 2025), including pathway databases (Wu *et al.* 2025). However, the need for a dedicated solution addressing molecular interaction maps is still missing. As these maps are highly interactive resources with a range of different applications, an LLM-based system should be developed and evaluated by directly engaging the target user group.

In this article, we describe a user-driven process of prototyping, development, and user testing of Llemy, an LLM-based system for exploring large pathway diagrams. Llemy is an online system built for exploratory analysis of user-built repositories of molecular interaction maps. We evaluated Llemy’s usability and its perceived performance by having a group of users conduct an exploratory analysis of various molecular interaction maps. We gathered information on the system’s performance at the level of individual user prompts and collected overall user feedback via a survey completed after their work. Based on these results, we identify key tasks performed by the users, and assess their perceived performance. We demonstrate a need for a robust framework for open-ended evaluation of tasks involving interactive diagram exploration and analysis. The results of this evaluation provide insight into the design of future LLM-based systems for exploring and analyzing molecular interaction maps and indicate the types of tasks on which such systems may underperform.

This article is structured as follows. In Section 2, we introduce the hackathon in which Llemy was prototyped, the user-driven approach to its development, its architecture, and the evaluation process. In Section 3, we briefly describe the user interface, discuss use case examples, and analyse user feedback data. Finally, we discuss the results and provide an outlook for future work.

Llemy can be accessed via https://llemy.vhp4safety.nl, and its code repository is available at https://github.com/ontox-project/Llemy (see Data Availability).

## 2 Methods

Llemy was developed in several steps, following a user-centric design. First, a prototype was developed during a two-day hackathon. This prototype was then refined and evaluated by a group of users involved in the development and analysis of molecular interaction maps.

### 2.1 Prototype development

The project was initiated during a two-day hackathon held at the University of Liège, Belgium, on 8–9 May 2025. The aim was to design a system that facilitates exploration of molecular interaction maps, with liver lipid and bile metabolism (Ladeira *et al.* 2025) as use cases. These maps were developed as a part of the ONTOX project (Vinken *et al.* 2021). The hackathon group involved hepatotoxicologists, curators of the maps, computational biologists, and LLM experts.

User requirements and the system architecture were established based on user prompts prepared by the domain experts. These prompts were then used in iterative prototyping and evaluation. The prompts included (i) queries with scope beyond the contents of the map and (ii) queries containing false premises, designed to evaluate the ability to identify and address incorrect assumptions (see Supplementary material H1).

The final hackathon prototype consisted of two separate agents collecting information in parallel: one from the map hosted on the MINERVA Platform, and one from Perplexity using deep research (accessed on 8 May 2025). Then, a third synthesis agent (OpenAI GPT4.1) combined and summarized the findings. Commercial LLMs were chosen for quick prototyping. The whole prototype was containerized in a Docker application with the possibility to deploy it locally and provide API keys for both LLMs. This architecture was inspired by the O-QT assistant (Djidrovski *et al.* 2026).

### 2.2 System architecture

Llemy was built entirely in Python, using GPT-4.1 nano, Streamlit (v1.50.0) for the front-end, and LangChain (v0.3.27) as the agentic backend. Note that it is possible for a user to change the model to another LLM in the OpenAI family, replacing MODEL_NAME=“gpt-4.1-nano” by their LLM of choice in workflow.py (line 23). Llemy is deployed publicly on a cloud platform (https://llemy.vhp4safety.nl/), and works with user-provided API keys. It is also available as a Docker container for local deployment. In the deployed version, API keys are stored only for the duration of a user session to ensure security. To avoid context rate limits, a follow-up prompt using the previous answer is not possible in the current version as of the publication of this article. However, the entire chat history from a given session is visible. Llemy combines user prompt with map data and instructions output, focus, and inclusion of literature references. The full instructions are available in the file workflow.py on the GitHub repository (https://github.com/ontox-project/Llemy).

### 2.3 User study design

Llemy was evaluated by a group of 25 users recruited via Disease Maps Community channels (https://disease-maps.io/). The users worked with the system by performing queries to a selected pathway or disease map, using either a pre-constructed set of prompts (see Supplementary material U1), or prompts provided by the users. Users were provided with API keys and asked to evaluate the system’s performance after each prompt. Performance metrics were accuracy, conciseness, and reliability, evaluated on a 5-point scale. Users were also able to provide free-text comments on the system’s performance for each metric. The API keys for user testing were provided by ELIXIR Luxembourg Node. At the end of their testing session, the users were asked to fill out a questionnaire summarizing their overall assessment of the performance of Llemy (see Supplementary material U2).

Two separate datasets were collected for analysis. One consisted of individual prompts, system replies, and user feedback on the accuracy, conciseness, and reliability of the responses (Prompt Dataset). The other dataset contained user replies to the final questionnaire (Summary Dataset). They were treated as separate in downstream analyses.

#### 2.3.1 Prompt dataset analysis

The Prompt Dataset contains 157 entries, including the user prompt, the response of the system, the response time, and the evaluation of the response in terms of accuracy, conciseness, and reliability, rated on a 5-point scale (see Supplementary Table U3). One prompt (‘“Describe how I can bake an apple cake”’) was intentionally designed to test the system’s ability to remain within the scope of its source maps. The system correctly responded by explaining its own purpose, and this entry was removed from the subsequent analysis as irrelevant.

To understand the prompt landscape, 114 unique prompts were categorized into three categories. The categories were established post hoc based on the content of the prompts. Each prompt was assigned a category based on a consensus among three independent experts. Score distributions were summarized, both aggregated across all categories and stratified by prompt category, to examine potential variation in perceived system performance across different categories. Free-text comments provided for each prompt evaluation were grouped by associated metric score and analyzed qualitatively. There were no comments for conciseness provided by the users.

#### 2.3.2 Summary dataset analysis

The summary dataset gathers a general evaluation of the system from 19 unique users. The data collection was fully anonymous. Users were asked about their experience using molecular interaction maps and LLMs. They were then asked to estimate how long they spent testing the app, and what type of prompts they entered. Further feedback on ease of use, productivity, and overall assessment was collected using both a 5-point scale and free-text comments.

### 2.4 Statistical analysis

All analysis was performed in R version 4.5.2. To analyse the impact of response time on performance metric, a cumulative link mixed model (ordinal package, clmm method, link = ‘probit’, nAGQ = 10) was fitted on accuracy, conciseness, and reliability metrics (jointly), with a random intercept per user to account for within-user correlation. A cumulative link mixed model was chosen because of high inter-metric correlation: Spearman correlation between accuracy and conciseness is 0.53, between conciseness and reliability is 0.43, and between accuracy and reliability is 0.71.

To examine differences in accuracy, conciseness, and reliability across categories, *post hoc* pairwise comparisons, using the FSA package, were conducted using Dunn’s test with Holm correction for multiple comparisons. This non-parametric test was applied separately for each dependent variable to identify which specific category pairs differed significantly while controlling for family-wise error rate.

## 3 Results

### 3.1 Implementation of Llemy

Llemy consists of two parallel processes (Fig. 1) following the prompt provided by the user, and the selected map. First, the user prompt is enriched with instructions for the LLM ensuring contextualised output, scientific focus, and inclusion of literature references (see Supplementary material P1). Second, a retrieval agent uses the MINERVA API (Hoksza *et al.* 2019) to get contents of the map selected by the user: elements (nodes), reactions (edges), and annotations. The system provides access to public maps available under a permissive licence shared via a repository of molecular interaction maps, MINERVA Net (https://minerva-net.lcsb.uni.lu/) (Gawron *et al.* 2023), and a set of predefined prompts. The contents are concatenated into a single text object, including element identifiers, reaction types, and identifiers of supporting literature. Importantly, this transformation removes SBML properties from analyzed diagrams to narrow the context of the LLM for more accurate answers. Because of this, downstream computational workflows would have to retrieve MINERVA identifiers provided by in the system response, and use them with MINERVA API calls to access full content of the map of interest,

**Figure 1:**
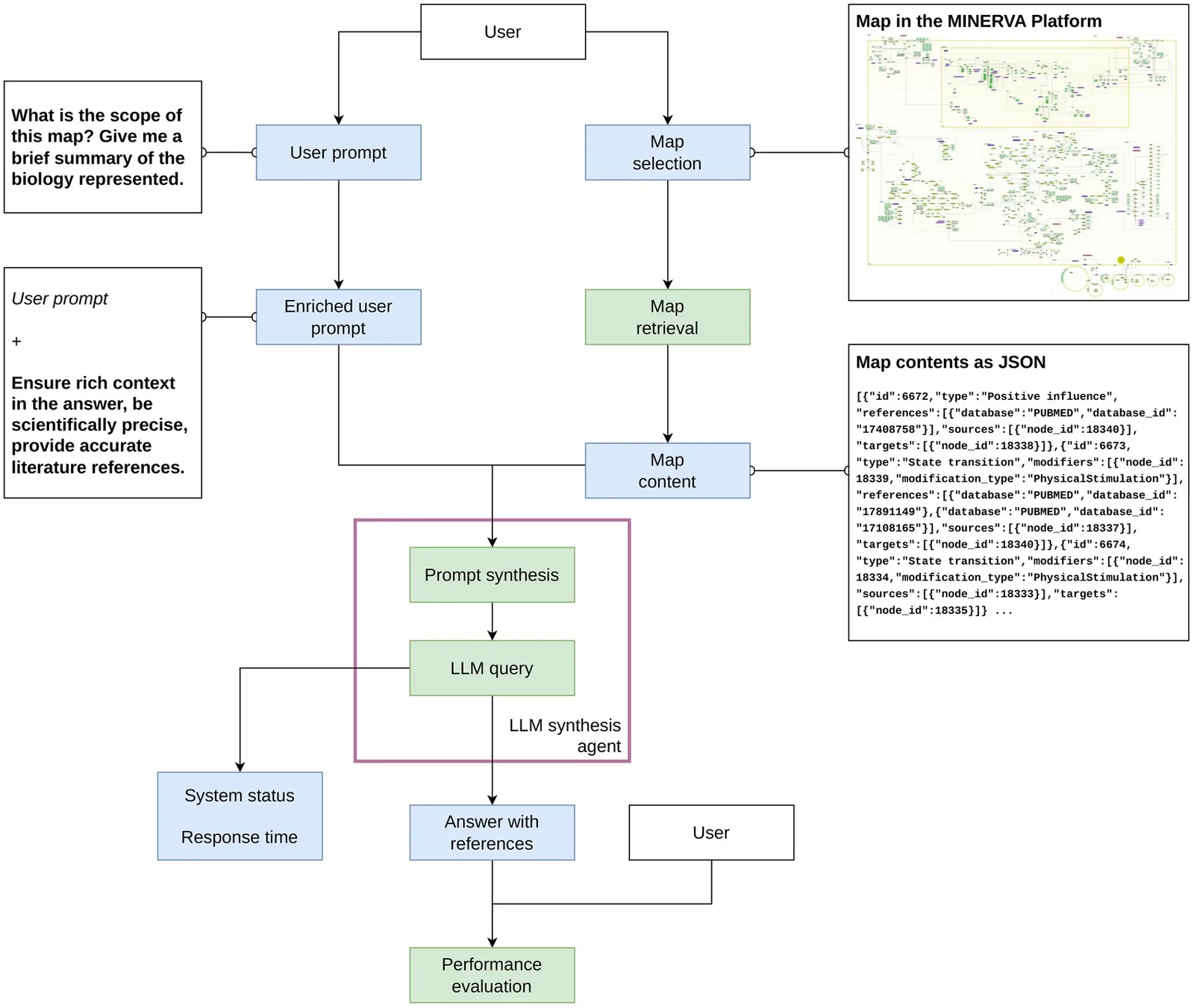
The workflow of Llemy. Users provide their prompt and select a map they want to process. Then, the prompt is enriched with instructions for the content of the answer (see full system prompt in Supplementary P1), and, in parallel, map data is fetched from the corresponding MINERVA Platform. Both enriched prompt and processed map data are combined and used as the LLM prompt. The answer is provided to the user for evaluation, and system performance logs are collected. Green: process, blue: data.

This text object is then given to a ‘synthesizer agent’ as a context, together with the enriched user prompt. The response is formatted *post hoc* by adding links to specific parts of the map of interest: when the LLM cites an element or reaction identifier in a given sentence, they are isolated and collated to a clickable link after the end of that sentence. A robust error-handling and logging layer is integrated throughout the workflow: API responses are normalized into explicit states (e.g. success, error, no_data_found, skipped), retrieval errors are passed to the synthesizer as structured context, and both the synthesized response and raw map payload are returned for traceability. This makes each answer auditable and allows users to verify exactly which MINERVA-derived data supported the final output. Response times for each query were also calculated.

Llemy is deployed as a service on the VHP4Safety cloud (https://llemy.vhp4safety.nl/). Upon landing on the page (see Fig. 2), the user is prompted to enter an OpenAI API key. To provide user feedback, a password is required to ensure privacy, and consent to collect prompt and response data to analyse the performance of the system. If the user previously agreed to the recording of their data, a brief feedback form is displayed to rate the accuracy, conciseness, and reliability of the answer on a 1–5 scale, and provide a free-text comment on the result of a particular query. Llemy can be used without providing feedback.

**Figure 2:**
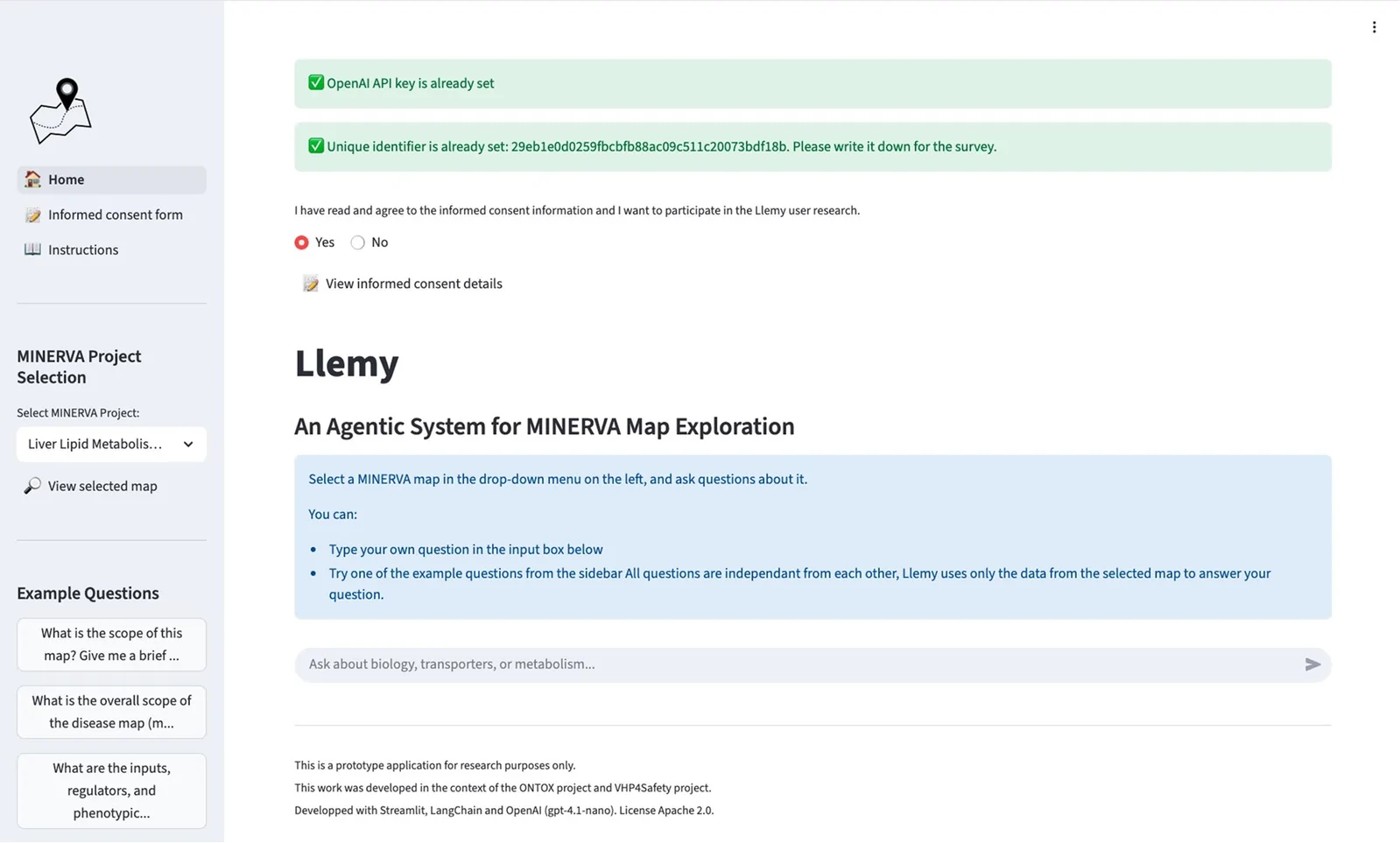
Screenshot of Llemy’s welcome page. The left-side panel comprises (i) a section with links to the home page, informed consent form, and user manual; (ii) a map selection drop-down menu, and a link to the selected map; and (iii) a list of example prompts (see Supplementary U1 for a complete list).

### 3.2 Use cases

The purpose of Llemy is to facilitate exploration of complex molecular interaction maps. Such maps can have thousands of elements and interactions, constructed from hundreds of literature references, which makes working with these resources a challenge, despite dedicated software supporting exploratory and analytical tasks. We identify three main groups of tasks that Llemy can facilitate when working with large molecular interaction maps. These are to (i) summarize large content into understandable reports, (ii) find specific elements, or (iii) analyse certain properties of the maps (see Table 1). Below, we discuss each of these categories based on concrete examples, which are available via Supplementary material E1. Each cited query below has a corresponding example file with a complete output of Llemy and the corresponding map from which the content was taken.

**Table 1:** Categories of prompts in the prompt dataset, and their short definitions.

Category	*N*	**%**	Definition
**Summarize**	65	41.7	Summary of a map, mechanisms involving specific pathways, elements, or interactions (e.g. autophagy, bile acid metabolism, signalling pathways), or mechanisms missing from the map
**Find**	46	29.5	Retrieval of specific map elements, elements with specific properties (e.g. kinases, ligands, enzymes, transcription factors, protein interactors), or upstream or downstream targets (e.g. receptors, organelles, phenotypes) outside the map
**Analyse**	45	28.8	Analysis of the map or its parts (e.g. count specific elements, calculate connectivity of the network) or reason about the consequences of perturbing the molecular network (e.g. drug effects, disease-related perturbations)

### 3.3 Summarize

These tasks support initial exploration of large maps by providing digestible reports about different aspects of the map. User queries may involve creating an overview of the contents of a given map to a new user, allowing to grasp key diagrams and pathways in a given map and evaluate its scope at a glance, e.g. ‘summarize the biology of COVID-19’ (summarise_6, Supplementary material E1). Another set of queries in this task asks for the role of a single molecule across different pathways, e.g. ‘summarize the role of NAMPT in ageing’ (summarise_1), which may gather map information about multiple instances of multi-context and multipurpose proteins in multiple map locations. Finally, queries about microenvironment, tissue specificity, temporal resolution, or inter-organelle interactions allow users to quickly understand the context of a given map and its utility for data interpretation and modelling, e.g. ‘summarize inter-organelle interactions, crossing e.g. nucleus, mitochondria, ER.’ (summarise_8).

### 3.4 Find

These tasks extend search functionalities of the MINERVA Platform beyond simple lookup of target molecules. LLM support offers natural language-based queries of the content of maps, which would be difficult to implement as a conventional search function. User queries may be similar to existing functionalities, e.g. ‘list all drugs in the map’ (find_2, Supplementary material E1), but Llemy supports advanced inquiries like ‘find stress pathways in the map’ (find_5), which requires a combination of the map contents, and the LLM reasoning of what may be considered a stress pathway. Finally, queries like ‘find the initial event for COVID pathology, and its endpoint’ (find_7) or ‘find the inputs, regulators, and phenotypic outputs’ (find_6) allow users to identify map elements that are functionally related to each other without time-consuming tracking of diagram connectivity.

### 3.5 Analyse

These tasks support reasoning and calculation-related queries, offering computational support over the content of molecular interaction maps. Given the limitations of LLM-based reasoning in some models, such support may have a moderate extent; nevertheless, it allows basic hypothesis building and may give users a better understanding of the modelling potential of a map, and an inspiration to construct advanced computational workflows. Queries like ‘identify pathways affected by blocking autophagy; explain your findings’ (analyse_2, Supplementary material E1) or ‘define measurable downstream changes of NAMPT attenuation; propose the best biomarker of NAMPT perturbation’ (analyse_4) are essentially modelling questions, however as the input that Llemy operates on is reduced, results of such queries should be followed by dedicated, reproducible computational workflows.

### 3.6 User study

#### 3.6.1 User feedback for individual prompts

We assessed the perceived performance of the system across three metrics—accuracy, conciseness, and reliability—based on user feedback (see Fig. 3). Medians for these three metrics were 4, 3, and 4, respectively. We performed a cumulative linked mixed model (see Section 2) to check if any of these metrics were affected by the response time. Response time had a significant negative effect on ratings (*β* = −0.34, SE = 0.06, *z* = −6.06, *P* < .001), indicating that longer response times were associated with lower perceived quality. This effect was uniform across accuracy, conciseness, and reliability. On average, users rated conciseness significantly lower than accuracy (*β* = −0.37, *P* = .002), while reliability did not differ from accuracy (*β* = −0.12, *P* = .34).

**Figure 3:**
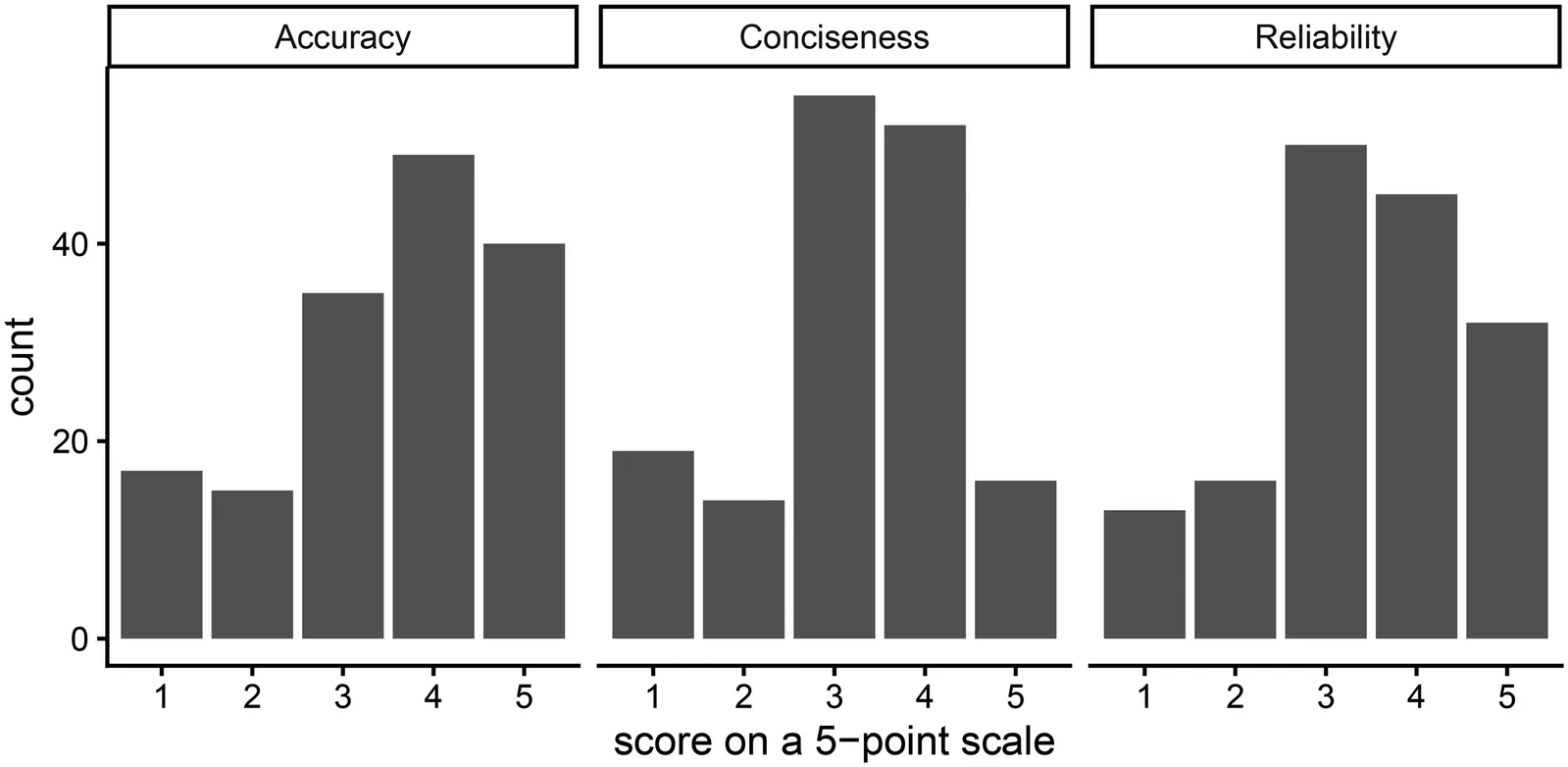
Score distribution by evaluation criterion.

Individual prompts were grouped into three categories (see Section 2), as shown in Table 1, together with the number of prompts per category. Stratified analysis of evaluation metrics using *post hoc* Dunn test (see Section 2), revealed no significant performance differences across categories. Mean accuracy, conciseness, and reliability were the highest for the ‘Summarize’ task (Fig. 4), and the ‘Find’ category exhibited a broader distribution of scores, showing lower evaluations for all scores, possibly reflecting the complexity of querying molecular interaction maps with an LLM when the content of the map is in the prompt.

**Figure 4:**
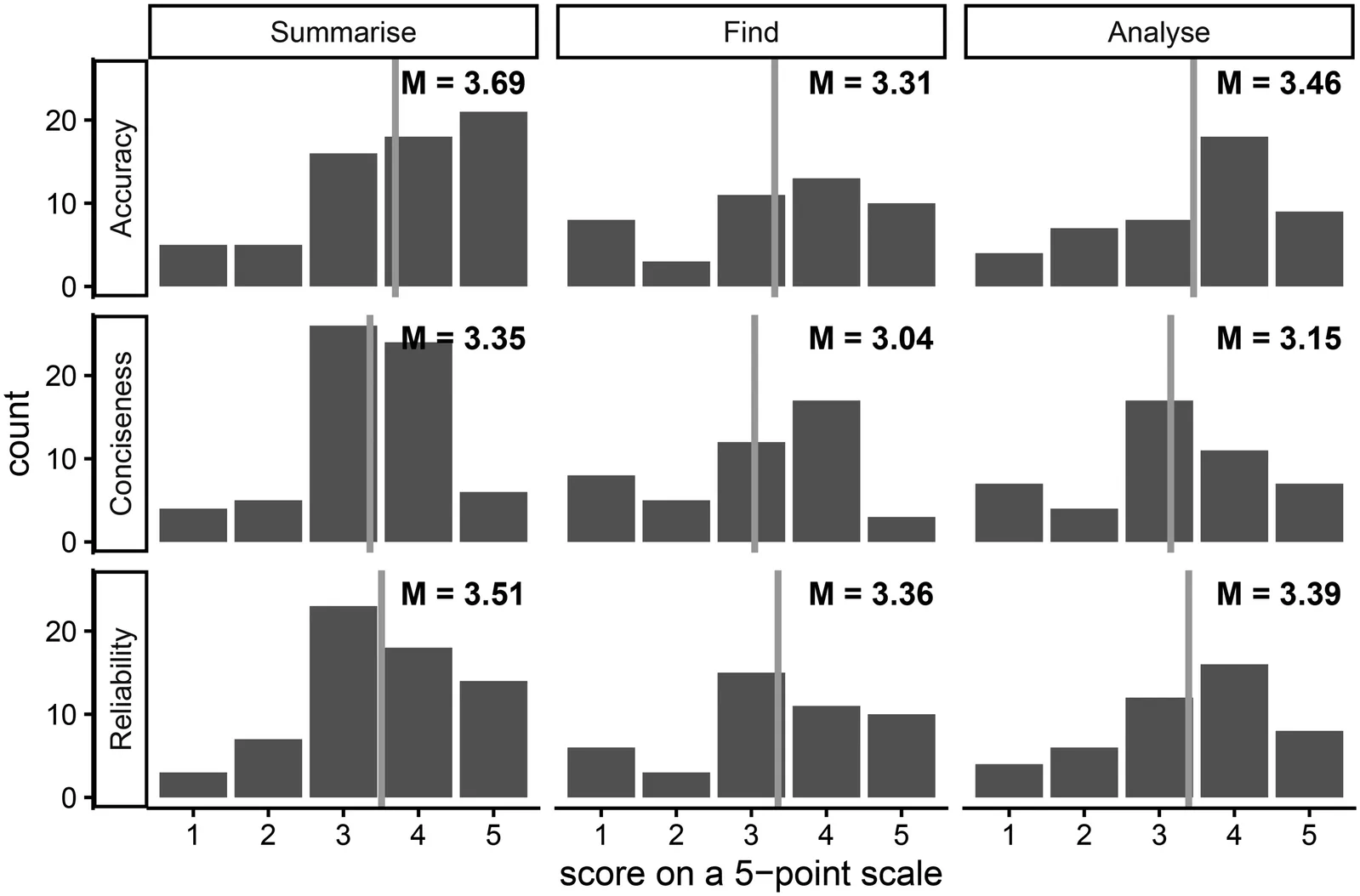
Distribution of tasks performed by the users testing Llemy, grouped into three categories. M, average.

Qualitative analysis of individual user comments revealed distinct patterns associated with score levels. For higher accuracy scores (4–5), users acknowledged the system’s ability to provide comprehensive summaries, correctly identify pathway connections across submaps, and appropriately acknowledge limitations when specific entities were absent from maps. For lower accuracy scores (1–2), users most frequently cited fundamental factual errors, failure to locate existing map content, and fabricated or mismatched map reaction references. Common criticisms included misunderstanding map element types, missing available annotations, and instances where the system failed to recognize entities present under synonyms. There were no individual comments on the conciseness of results. Reliability scores were strongly influenced by the quality of the reaction hyperlinks to the source content, coming from maps hosted in the MINERVA platform. High reliability scores (4–5) corresponded to systematic and verifiable referencing, allowing systematic verification. Low reliability scores (1–2) were associated with broken or non-resolving links and inconsistent behaviour across repeated queries, notably varying structure and scope of provided references to the source map, from insufficient to unnecessarily repetitive.

Several cross-cutting themes emerged from user feedback. First, synonym and nomenclature handling presented recurring challenges when the system failed to recognize entities represented by HGNC (HUGO Gene Name Committee) names rather than common abbreviations. Second, users valued contextual awareness, noting that responses sometimes missed map-specific or domain-specific framing (e.g. failing to mention organ context for an organ-specific map). Third, users at intermediate score levels (3) noted that answers were accurate but incomplete, and challenging to assess due to a lack of proper references to the source material.

Detailed, representative examples of queries where Llemy underperformed are available as described in Supplementary material E1, ‘challenging-cases’. Summarized individual user comments having particular score levels are listed in Supplementary material U4.

#### 3.6.2 Overall user feedback

The user survey showed a varied distribution of users, curators, and developers of molecular interaction maps. The relatively low number of responses in the ‘user’ group can be explained by the fact that most users also had experience as curators or developers. Almost all users had prior experience with LLMs. All users tested the system for more than 15 min, half of them spent more than an hour, and 75% estimated that they saved time by using Llemy. Another important feedback was concerning the variability of the output for similar queries, where, on a 1–5 scale, all users except one reported variability of 3 and higher. Figure 5 summarizes usefulness and usability scores based on experience with molecular interaction maps.

**Figure 5:**
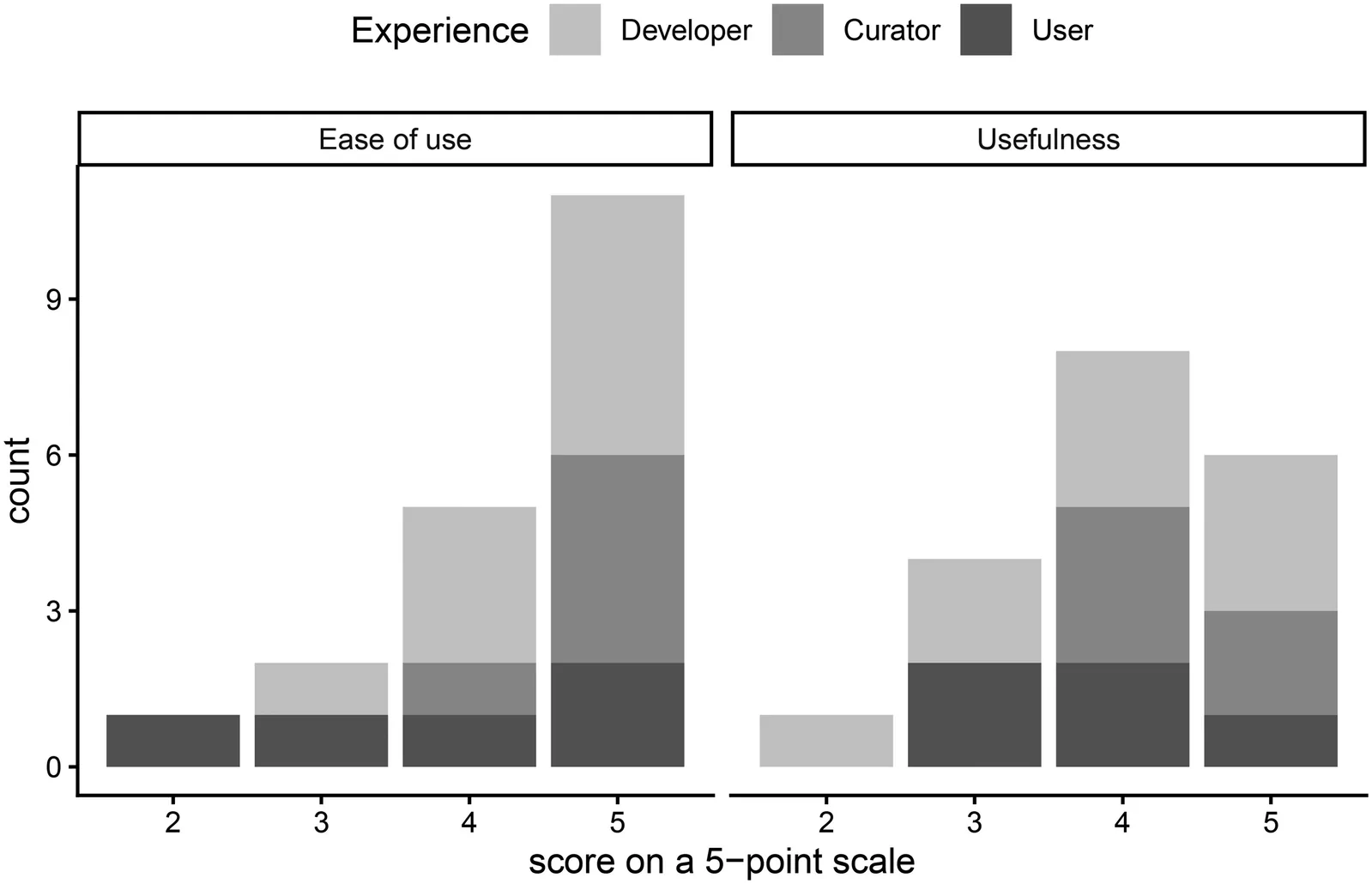
Usefulness and usability based on experience with molecular interaction maps. Developer—participants building workflows including maps; curator—participants creating maps; user—participants using the maps for knowledge retrieval and data visualization. in case of multiple roles, the highest level of experience—developer, then curator, then user—is taken into account.

## 4 Discussion

### 4.1 Advantages of the LLM-based approach

The potential of LLMs to accelerate research in the field of molecular pathways and their regulation is a topic of intense investigation. In this work, we explore the capability of LLM-based systems to process, summarize, analyse, and suggest extensions of structured, focused knowledge repositories describing molecular pathways involved in human physiology and pathology. We introduce Llemy, a system for summarizing knowledge graphs based on user prompts. Our approach parallels previously published LLM solutions to refine knowledge graphs from literature (Feng *et al.* 2025, Khan *et al.* 2025, James *et al.* 2026), However, in the present work the sources of the system are manually curated diagrams. This allows Llemy to directly reference diagram elements and interactions, thereby improving the system’s transparency and facilitating exploration of the original content based on the system’s response.

### 4.2 User-focused analysis

Our approach focused on the user perspective in the design and evaluation of the system. The system was designed and prototyped during a hackathon, when domain experts directly formulated prompts they would ask, and evaluated replies on-the-fly. The finalized system was tested by an independent group of 25 users at the level of individual prompts, and via a survey regarding their overall user experience. Based on 156 individual prompts, we proposed three categories posed to the system and showed that tasks with higher complexity are asked less frequently and receive less positive feedback. Although no statistical differences were observed, we have noticed comparatively higher scores in the ‘Summarize’ category. Based on these categories, Llemy workflows can be tailored to users needs. Thus, the present work parallels the functionality of the Alvessa system proposed in (Sokolova *et al.* 2025), but it focuses on the nature of the task rather than on specific resources to be queried based on the system prompt.

One of our findings was a negative impact of response time on performance metrics, i.e. longer response times corresponded to worse performance. Notably, literature reports in this regard are mixed (Hu *et al.* 2025, Maslych *et al.* 2025, Wang and Lo 2025, Kim *et al.* 2026), so our results should not be overinterpreted. Importantly, specific examples of Llemy output for the same query, for the same map (see ‘variability’, Supplementary material E1) indicate multiple factors impacting varying response time. In highlighted examples, Llemy answers differ in length, content, and structure, but their length is not correlated with the response time. In one of the answers, the system provides a link to some related proteins in the map, but not the others. In some examples, no links are provided. In this light, longer response times can be explained by the impact of MINERVA API when providing the map or variable response time by the LLM, but it is unlikely that it correlates with the complexity of the answer.

### 4.3 User perception of an LLM-based system

A summary user survey completed by 19 of 25 participants rated the system as highly accessible; however, the utility rating was more moderate, particularly among map users. This may be explained by the fact that the users less familiar with the content processed by Llemy can give more favourable evaluations to its answers. All users generally rated the usability of Llemy as high (see Fig. 5), with over 80% rating it a 4 or 5. It is worthwhile to note that users-only were quite split. Over 70% of users found Llemy useful (score of 4 or more).

Reproducibility of results and the sustainability of the system are key considerations in developing systems such as Llemy. All users except one reported a marked variability of the system output, of at least 3 points on the 1–5 scale, in line with other reports (Wang and Wang 2025). To improve this outcome, open-weight models may be considered, contrary to the current commercial LLM. Open-weight LLM models show promising overall performance when compared to commercial solutions (Stachura *et al.* 2025), however, use of open LLMs comes with the need for a dedicated infrastructure and associated costs. Moreover, tests of locally deployed, open-access, and open-weight LLMs are necessary to compare the tradeoff between quality and cost of use, which in turn requires benchmark datasets. Our open-ended user survey may inform the design of such a system, similar to foundational model benchmarks (Laurent *et al.* 2024) used in Sokolova *et al.* (2025).

### 4.4 Roadmap for the system and its evaluation

Llemy and similar LLM-powered systems have the potential to reduce the complexity barrier when working with complex diagrams. Based on the user feedback in both the Summary and Prompt Datasets, we drafted a roadmap to support future improvements. We attributed priorities based on the frequency with which users requested the feature. In the short term, systems architecture should be updated to improve response time and accuracy of the provided references. Afterwards, dedicated workflows for summarizing, querying, and analyzing the map should be introduced, with task granularity matching the user study. Finally, programmatic integration of Llemy functionalities with the GUI of the MINERVA Platform using plugins (Hoksza *et al.* 2019) and workflows using the Model Context Protocol (Hou *et al.* 2025, Kuehl *et al.* 2025) will streamline the interaction of LLMs with the content of maps. This should be supported by the use of open-weight LLMs to address the needs of the open-access research environment.

User interaction is an important part of the Llemy project, and it can be improved by acting directly on provided feedback. Analysis of individual user prompts and answers, especially for the low-scoring cases (see ‘challenging-cases’, Supplementary material E1) provides a valuable window into improvements needed for the system. First, we need to improve input limitations that cause errors where requested content is inaccessible directly from the prompt, like specific identifiers or synonyms. Second, we have to address reasoning limitations, likely related to the chosen model, which may cause Llemy to stumble on multi-step problems, like connecting a synonym to its official symbol and then looking for that symbol in the content of the map. Finally, we need to fix mishandling of MINERVA references which causes repetitive artifacts in the output.

These issues can be addressed by supporting term disambiguation and feedback for prompts that cannot be answered, as well as better linking of references to original publications or map content used in the answer. External services for grounding biomedical terms (James *et al.* 2026) can assist in this task. Better transparency into requested data and its use in answering user queries will improve understanding and evaluation of the system’s behaviour. These steps will lead to an improved user evaluation system, likely combining benchmarks specific to proposed tasks with an open-ended scoring system.

The user-based evaluation system applied for Llemy requires refinement and further development, together with the changing landscape of technology and its possible applications. Open-ended user testing in computational biology is a limited, but expanding area (Mitchener *et al.* 2025), which can inform the development of highly interactive and multipurpose resources like molecular interaction maps. The framework proposed in this article is limited to three metrics of performance, which nevertheless allowed us to *post hoc* identify main tasks explored by the user community. This informed the roadmap of Llemy development, and can be expanded into a set of task-specific evaluations, combining structured and open-ended scenarios of map exploration and analysis. Evaluators from communities of interest should be iteratively engaged when such refinements become available, including pathway and network interaction databases, like Reactome (Ragueneau *et al.* 2026), WikiPathways (Agrawal *et al.* 2024), or SIGNOR (Lo Surdo *et al.* 2026), and synergizing with relevant efforts (Mohammadi *et al.* 2025). The timeline of future user testing depends on system development, but can be supported by an engaged community, tailoring LLM support to user needs.

### 4.5 Study limitations

Our study faces a number of limitations. First, the limited number of users and the recruitment process may have affected the survey’s overall evaluation and conclusions. This can be seen in the relatively low number of participants in the ‘user’ group, i.e. people with no prior knowledge about the development or analysis of molecular interaction maps. Second, Llemy features an inconsistent output, which affects the evaluation by its users. The answers vary for prompts having the same content and structure, including missing references, varying answer size, or overwhelming and unnecessary repetitions. This is a known behaviour of LLM-based systems for complex tasks (Wang and Wang 2025), and is beyond the scope of our current work. Next, due to technical limitations, the size of the content processed by Llemy is restricted by the LLM capabilities, resulting in a limited scope of user queries. A potential mitigation for this issue would be to move from a long-context architecture to a (graph) retrieval-based approach, using solutions akin to those developed by Lobentanzer *et al.* (2025). Finally, since LLM providers continuously update model behaviour, the results should be interpreted as a snapshot of the tested model, its version and evaluation period. A model change may affect answer quality and scope. Llemy is designed so the underlying LLM can be exchanged, however any substantial model change should trigger re-evaluation using the same framework before. Use of GPT-4.1-nano was a pragmatic choice for a user-facing prototype because of its low latency and lower cost use. Future work should compare other models to quantify the trade-off between speed, cost, and response quality.

## 5 Conclusions

Llemy shows a promising use case for the usage of LLMs to interact with biological databases, particularly as a summarization tool for an entry point into molecular interaction maps. The user-driven approach we took is important for evaluating the needs and perception of LLM-based systems in science, and yielded valuable insights on further development, in particular for more advanced tasks. With a rapidly changing landscape of available LLMs, it is important to construct sustainable systems that can be benchmarked when underlying models change. User-driven development and open-ended benchmarking systems can support these LLM comparisons, especially for complex tasks using interactive tools. Future plans also include re-running the study with an extended framework, as well as recruiting more uniformly distributed participants for a more generalizable evaluation and a better coverage of possible tasks.

## Supplementary Material

vbag277_Supplementary_Data

## Data Availability

Llemy’s code and documentation are available at GitHub (https://github.com/ontox-project/Llemy), under the Apache 2.0 License (https://www.apache.org/licenses/LICENSE-2.0). A public instance is hosted at https://llemy.vhp4safety.nl/.
