# Supplementary material for: User-driven development and evaluation of an agentic framework for analysis of large pathway diagrams
Source: Bioinform Adv. 2026 Sep 21;6(1):vbag277. doi: 10.1093/bioadv/vbag277 (PMC13674548; doi:10.1093/bioadv/vbag277)
Supplement: Supplementary file 1 [file vbag277_supplementary_data.zip › Supplementary E1.pdf]

Supplementary E1: Llemy examples

Llemy github repository contains a list of query-answer examples to illustrate the behaviour and functionality of the system. The “examples” subdirectory https://github.com/ontox-project/Llemy/tree/main/examples contains the following subdirectories:

use-cases
This subdirectory contains examples of Llemy responses for three main task categories, Analyse, Find, and Summarise (see Table 1 of the manuscript).

Each example file contains:
- Accessed map: what map was processed
- User query: what the user requested, redacted to remove potentially sensitive information
- Question category: what task category this query was assigned
- Response time, in seconds, rounded
- Answer from the system

Index of use case examples

example	user query	link
analyse_1	Identify drugs that mimic the effects of rapamycin, e.g. affecting another pathway with a similar function.	https://github.com/ontox-project/Llemy/blob/main/examples/use-cases/analyse/analyse_1.md
analyse_2	Identify pathways affected by blocking autophagy. Explain your findings.	https://github.com/ontox-project/Llemy/blob/main/examples/use-cases/analyse/analyse_2.md
analyse_3	Does the map support a timeline for pathway activation in normal aging?	https://github.com/ontox-project/Llemy/blob/main/examples/use-cases/analyse/analyse_3.md
analyse_4	Define measurable downstream changes of Nampt attenuation. Propose the best biomarker of Nampt perturbation.	https://github.com/ontox-project/Llemy/blob/main/examples/use-cases/analyse/analyse_4.md
analyse_5	How many diseases are in the map?	https://github.com/ontox-project/Llemy/blob/main/examples/use-cases/analyse/analyse_5.md
analyse_6	Identify clinical effects of increased Bacteroides thetaiotaomicron.	https://github.com/ontox-project/Llemy/blob/main/examples/use-cases/analyse/analyse_6.md
analyse_7	Identify and provide key sentinel nodes in the map.	https://github.com/ontox-project/Llemy/blob/main/examples/use-cases/analyse/analyse_7.md
analyse_8	Identify triggers and drivers of the pathological response in this map.	https://github.com/ontox-project/Llemy/blob/main/examples/use-

		cases/analyse/analyse_8.md
find_1	List diseases included in this map.	https://github.com/ontox-project/Llemy/blob/main/examples/use-cases/find/find_1.md
find_2	List drugs included in the map.	https://github.com/ontox-project/Llemy/blob/main/examples/use-cases/find/find_2.md
find_3	Find PubMed ID for RID: 55717.	https://github.com/ontox-project/Llemy/blob/main/examples/use-cases/find/find_3.md
find_4	Find all receptors present on the map.	https://github.com/ontox-project/Llemy/blob/main/examples/use-cases/find/find_4.md
find_5	Find stress pathways in the map.	https://github.com/ontox-project/Llemy/blob/main/examples/use-cases/find/find_5.md
find_6	Find the inputs, regulators, and phenotypic outputs.	https://github.com/ontox-project/Llemy/blob/main/examples/use-cases/find/find_6.md
find_7	Find the initial event for COVID pathology, and its endpoint.	https://github.com/ontox-project/Llemy/blob/main/examples/use-cases/find/find_7.md
find_8	Find stress pathways (DNA damage, ER stress, oxidative stress, unfolded protein response) in the map.	https://github.com/ontox-project/Llemy/blob/main/examples/use-cases/find/find_8.md
summarise_1	Summarise the role of NAMPT in ageing.	https://github.com/ontox-project/Llemy/blob/main/examples/use-cases/summarise/summarise_1.md
summarise_2	Summarise tissue- or cell-type specificity. Indicate multiple tissues or cell-types represented.	https://github.com/ontox-project/Llemy/blob/main/examples/use-cases/summarise/summarise_2.md
summarise_3	Summarise temporal aspects - early and late disease stages - in the map.	https://github.com/ontox-project/Llemy/blob/main/examples/use-cases/summarise/summarise_3.md
summarise_4	Summarise a disease or diseases represented in the map.	https://github.com/ontox-project/Llemy/blob/main/examples/use-cases/summarise/summarise_4.md
summarise_5	Summarise tissue- or cell-type specificity. Indicate multiple tissues or cell-types represented.	https://github.com/ontox-project/Llemy/blob/main/examples/use-cases/summarise/summarise_5.md
summarise_6	Summarise the biology of COVID-19.	https://github.com/ontox-project/Llemy/blob/main/examples/use-

		cases/summarise/summarise_6.md
summarise_7	Summarise the role of the microenvironment - inflammatory, metabolic - in modulate key pathways.	https://github.com/ontox-project/Llemy/blob/main/examples/use-cases/summarise/summarise_7.md
summarise_8	Summarise inter-organelle interactions, crossing e.g. nucleus, mitochondria, ER.	https://github.com/ontox-project/Llemy/blob/main/examples/use-cases/summarise/summarise_8.md

variability

Examples provided here illustrate variable output of Llemy from the same map, Liver Lipid Metabolism Map of the ONTOX project (https://ontox-project.eu/). The answer is based on the same user question, one of the default options supplied by Llemy: `What is the scope of this map? Give me a brief summary of the biology represented`.

challenging-cases

These examples describe cases where Llemy underperformed and got low scores (1 or 2) on at least two out of three performance metrics.

Each example file contains:
- Accessed map: what map was processed
- User query: what the user requested, redacted to remove potentially sensitive information
- Metrics: which metrics were indicated as low by users
- User comment: written feedback from a given user, redacted to remove potentially sensitive information
- Developer comment: feedback and comment of Llemy developers providing further detail and speculation about the reasons for this particular behaviour

Index of challenging case examples

**example**	**user query**	**link**
challenging_1	Find chemical identifiers, specifically the InChIKey, for map reactions.	https://github.com/ontox-project/Llemy/blob/main/examples/challenging-cases/challenging_1.md
challenging_2	Does the map contain DAT - the dopamine transporter? Indicate processes is it linked to and in cell types it is active in.	https://github.com/ontox-project/Llemy/blob/main/examples/challenging-cases/challenging_2.md
challenging_3	Summarize the role of DNMT1 in this map.	https://github.com/ontox-project/Llemy/blob/main/examples/challenging-cases/challenging_3.md
challenging_4	What it the end outcome of the pathway in keratinocyte?	https://github.com/ontox-project/Llemy/blob/main/examples/challenging-cases/challenging_4.md
challenging_5	How many reactions are in the map?	https://github.com/ontox-

		project/Llemy/blob/main/examples/challenging-cases/challenging_5.md
challenging_6	How does the microbiome contribute to PD?	https://github.com/ontox-project/Llemy/blob/main/examples/challenging-cases/challenging_6.md
challenging_7	Indicate 10 key reactions of the map	https://github.com/ontox-project/Llemy/blob/main/examples/challenging-cases/challenging_7.md
challenging_8	List phenotypic nodes present in the map.	https://github.com/ontox-project/Llemy/blob/main/examples/challenging-cases/challenging_8.md

prompt
This subdirectory contains a file with a complete input to Llemy, including the content of the map transformed for the needs of Llemy prompt.
