# Supplementary material for: User-driven development and evaluation of an agentic framework for analysis of large pathway diagrams
Source: Bioinform Adv. 2026 Sep 21;6(1):vbag277. doi: 10.1093/bioadv/vbag277 (PMC13674548; doi:10.1093/bioadv/vbag277)
Supplement: Supplementary file 1 [file vbag277_supplementary_data.zip › Supplementary U3.pdf]

Supplementary U3: User evaluation metrics

ID	Category	Response time (seconds)	Feedback accuracy score	Feedback conciseness score	Feedback reliability score
001	Analyse	28.2163	5	3	4
002	Summarise	22.4099	2	1	2
003	Find	78.1817	5	4	5
004	Find	222.0802	4	2	5
005	Find	412.6278	2	3	2
006	Summarise	38.4972	5	4	5
007	Analyse	33.1146	5	4	3
008	Summarise	42.2872	3	3	3
009	Analyse	43.8341	4	3	3
010	Find	81.2427	3	3	3
011	Analyse	21.3286	5	1	5
012	Analyse	21.3286	5	2	5
013	Summarise	42.8327	3	3	3
014	Summarise	225.4019	1	3	3
015	Find	35.3072	4	3	4
016	Find	456.1268	1	1	1
017	Out of Scope	27.4588	4	3	5
018	Summarise	82.804	5	5	5
019	Summarise	21.235	1	3	1
020	Summarise	20.9294	2	3	2
021	Summarise	18.2458	3	3	3
022	Find	244.8034	3	1	1
023	Summarise	21.4183	5	1	5

024	Summarise	25.7209	5	4	4
025	Summarise	76.9444	5	3	5
026	Summarise	34.0399	4	4	4
027	Analyse	76.4653	1	2	2
028	Find	81.9757	5	5	5
029	Analyse	82.5072	4	2	1
030	Find	264.0319	4	2	4
031	Analyse	17.968	5	3	5
032	Find	18.5621	1	3	1
033	Analyse	28.3291	4	4	4
034	Summarise	35.8252	2	3	3
035	Analyse	151.7659	1	1	3
036	Analyse	237.554	1	1	3
037	Summarise	462.1365	1	1	1
038	Summarise	36.3324	4	3	4
039	Analyse	14.8958	4	4	4
040	Analyse	86.5625	2	4	2
041	Analyse	44.2533	5	4	5
042	Analyse	37.787	3	3	3
043	Analyse	78.5608	3	4	4
044	Analyse	74.8395	2	3	3
045	Find	44.6514	3	3	3
046	Analyse	20.4002	4	4	4
047	Analyse	77.1516	2	2	3
048	Analyse	33.0616	4	4	4
049	Summarise	36.3417	3	3	4
050	Find	26.4415	3	3	3
051	Find	30.4079	4	4	4

052	Summarise	30.0975	5	5	5
053	Analyse	19.6508	3	3	3
054	Summarise	84.2385	4	4	4
055	Summarise	78.545	2	2	4
056	Summarise	21.7004	5	1	5
057	Summarise	25.5644	5	1	5
058	Summarise	30.3399	4	4	4
059	Summarise	34.7802	5	4	5
060	Find	34.3063	3	4	4
061	Summarise	437.6224	1	2	1
062	Find	20.5333	3	1	3
063	Summarise	36.8527	2	3	2
064	Find	26.9604	4	3	1
065	Find	32.0679	3	3	2
066	Find	18.1711	4	3	3
067	Find	276.6109	1	1	1
068	Analyse	28.4058	5	4	3
069	Analyse	10.3914	1	1	3
070	Find	929.1113	4	4	4
071	Find	251.653	3	4	4
072	Analyse	28.3122	4	4	3
073	Summarise	31.1276	2	3	2
074	Find	44.8807	3	4	4
075	Summarise	23.1607	4	5	5
076	Find	35.0634	5	5	2
077	Analyse	26.3004	4	3	4
078	Analyse	35.4496	5	5	5
079	Analyse	39.4214	4	4	3

080	Analyse	29.2793	3	3	3
081	Summarise	239.7815	2	2	2
082	Find	27.2643	5	5	4
083	Find	3.7125	4	4	4
084	Summarise	36.7102	4	4	4
085	Analyse	6.1923	5	5	5
086	Find	191.712	4	2	4
087	Find	191.712	4	1	4
088	Analyse	20.777	5	5	4
089	Analyse	31.2476	5	5	5
090	Analyse	19.177	4	5	5
091	Summarise	34.0604	3	3	2
092	Summarise	228.1139	3	3	3
093	Analyse	232.7486	4	1	3
094	Summarise	37.6147	5	5	4
095	Summarise	37.6147	5	5	5
096	Summarise	34.4011	4	3	4
097	Summarise	36.9448	5	5	4
098	Summarise	32.5815	2	3	3
099	Summarise	35.5714	3	4	3
100	Find	35.7028	4	4	2
101	Find	34.8528	4	4	4
102	Find	34.3248	3	2	3
103	Find	23.3839	5	4	5
104	Find	28.2461	3	3	3
105	Find	13.7126	1	2	3
106	Find	22.5613	3	3	3
107	Find	20.2563	3	3	4

108	Analyse	14.3613	1	4	1
109	Find	19.7944	5	4	3
110	Summarise	76.0591	3	3	2
111	Summarise	25.227	4	4	4
112	Summarise	23.2739	3	3	3
113	Summarise	8.0092	5	4	5
114	Summarise	79.6202	2	3	3
115	Summarise	16.0143	3	3	3
116	Summarise	86.6634	5	5	5
117	Summarise	42.7544	4	5	3
118	Summarise	44.859	4	4	4
119	Summarise	21.3974	5	3	5
120	Summarise	33.542	4	4	4
121	Summarise	36.1631	4	3	4
122	Summarise	23.4826	1	2	3
123	Summarise	28.9572	4	3	3
124	Analyse	6.3152	4	4	3
125	Find	28.7738	5	4	5
126	Summarise	14.9326	2	2	3
127	Analyse	33.975	5	3	3
128	Analyse	90.2133	5	4	4
129	Analyse	40.8464	3	3	3
130	Summarise	34.2553	4	3	4
131	Analyse	225.6587	3	4	4
132	Analyse	156.689	3	1	3
133	Analyse	83.6239	3	4	3
134	Find	26.9125	4	3	4
135	Find	75.1455	4	3	4

136	Find	83.9212	4	4	2
137	Analyse	44.1013	4	3	3
138	Find	252.1148	1	1	1
139	Analyse	84.3028	5	3	2
140	Summarise	27.4738	4	4	4
141	Analyse	242.6845	1	1	1
142	Find	20.1116	3	3	2
143	Analyse	9.3164	4	4	4
144	Analyse	50.6857	5	4	5
145	Analyse	37.2194	3	4	3
146	Analyse	39.5466	4	4	3
147	Analyse	43.6011	5	4	5
148	Find	36.5658	4	4	5
149	Find	265.6204	1	1	1
150	Find	27.5797	4	4	4
151	Find	42.6832	5	4	5
152	Find	166.7365	4	4	3
153	Find	24.0201	3	4	4
154	Analyse	49.0015	5	3	5
155	Analyse	36.4246	2	3	3
156	Analyse	27.747	3	3	3
157	Summarise	26.991	5	5	5
