# Supplementary material for: User-driven development and evaluation of an agentic framework for analysis of large pathway diagrams
Source: Bioinform Adv. 2026 Sep 21;6(1):vbag277. doi: 10.1093/bioadv/vbag277 (PMC13674548; doi:10.1093/bioadv/vbag277)
Supplement: Supplementary file 1 [file vbag277_supplementary_data.zip › Supplementary U4.pdf]

Supplementary U4: Grouping of user’s comments by score ranking for Accuracy and Reliability

Accuracy:

- Score 1: Fundamental factual errors (incorrect statements about map contents, failure to locate existing entities, fabricated references);
- Score 2: Partially correct with significant errors (initial elements accurate but subsequent content flawed, failure to recognise map structure, fabricated supporting text);
- Score 3: Generally reasonable but incomplete (correct directionality with gaps in coverage, missed existing content under alternative nomenclature, difficult to verify without references);
- Score 4: Accurate with minor issues (correct information with small gaps, presentation or source attribution concerns, minor reference errors);
- Score 5: Excellent accuracy (comprehensive coverage of map content, appropriate adherence to source boundaries, transparent about limitations).

Reliability:

- Score 1: Broken links and unusable output (non-functional references, system errors, duplicate or malformed output);
- Score 2: Inconsistent results (variable quality across repeated attempts, links pointing to incorrect content, missing expected functionality);
- Score 3: Functional but inconsistent linking (arbitrary reference selection, excessive duplication, absent references limiting verification);
- Score 4: Generally reliable with minor gaps (correct but incomplete referencing, occasional link errors, generally appreciated when present);
- Score 5: Consistently accurate and well-referenced (correct source identification, transparent communication about coverage).
